# Food Science without Borders

**DOI:** 10.3389/fnut.2015.00033

**Published:** 2015-10-22

**Authors:** Dietrich Knorr, Chor San H. Khoo

**Affiliations:** ^1^Department of Food Biotechnology and Food Process Engineering, Technische Universität Berlin, Berlin, Germany; ^2^North American Branch of the International Life Sciences Institute, Washington, DC, USA

**Keywords:** food science, nutritional sciences, global food challenges, food chain integration, interdisciplinary research, transdisciplinary research, systems approach

The recent 25th anniversary of the fall of the Berlin Wall serves to remind us that sometimes “falling walls” are needed to solve differences not only between nations but also among the different disciplines of science. This need is underscored by a recent paradigm shift in research approaches to address multilayer and multistage research problems. This shift has seen a gradual transition from a “reductionist” approach to a more encompassing “total systems” approach in problem solving. One such approach is transdisciplinary research. Transdisciplinary research is currently widely used in biomedical and health programs and is gaining popularity in other areas as well, including climate change and environmental impact, food security, and sustainability (1–3).

Unfortunately, within biomedical research, the involvement of food and nutrition sciences is underemphasized in the transdisciplinary research equation. As a result, food and nutritional impact and its importance in health and disease management are understudied and under-recognized. This lack of representation can have long-term consequential effects for food research, impacting research funding, student recruitment and training grants, publication interest, impact realization, and prioritization (4).

For investigators in the food science, this trend is worrisome, as exemplified by the current emphasis on the impact and h-index factors to evaluate scientific performance, advancements, and funding. Areas that have been involved in biomedical and medical engineering with a health and disease focus (e.g., chemistry, biology, engineering, and medicine) have received increased funding attention, prioritization, and resources. This has led to a surge in researchers entering these research fields. In addition, it has also increased interest by research journals to publish in these areas.

Food and diet studies are complex and involve several scientific disciplines. Within the food-related fields, there are food technologists, food engineers, chemists, microbiologists, psychologists, psychophysiologists, biochemists, biologists, statisticians, sensory physiologists, toxicologists, nutritionists, and biotechnologists. Many food researchers have different societal affiliations, publication interest and limited interaction with each other. Matters are more complex in the nutrition fields, with different training emphasis among the various groups – nutrition science, dietetics, medicine, nutrition biochemistry.

Food research is often misunderstood as “soft” research, focusing primarily on technology development and application. On the contrary, many areas in food research (e.g., food safety and product efficacy, impact on health outcomes) require both basic and applied sciences. This knowledge is not widely known, and it is the only contributing reason why the food science discipline is often not included in biomedical research. In an example related to a national research grant review session, reviewers with medical backgrounds tended to underrate the scientific and research capability of food and nutritional experts or food engineers because of a perceived misconception that these scientists lack sufficient experience and participation in research projects or publication in high-impact journals.

There are other instances in which recognition of food science as a “hard” science has been under-recognized, despite the tremendous responsibilities and challenges to the field to provide safe, healthy, sustainable, and functional food, as well as safe drinking water and nutrition security to every human being. With the world’s population increasing from currently 7 to 9.6 billion by 2050 (5), there are tremendous challenges ahead. Some of the challenges include the emergence of new pathogens, an increasing aging population, the obesity pandemic, climate change, food and nutrient losses and waste, and hunger (6). Solving these issues require the collective attention of all related disciplines along the food chain. This calls for a need to take down the interdisciplinary walls among scientists and to work closely on productive solutions to achieve food and nutrition security (7).

At a workshop of the 2014 World Congress of the International Union of Food Science and Technology, young investigators of food science identified the critical need for increased communication among scientists, policymakers, governments, non-governmental organizations, and consumers (8). It is crucial to follow such suggestions from the future leaders in food science. In addition, the involvement of international public and private funding bodies is also critical.

One encouraging example of how multidisciplinary cooperation has worked is the European Union-founded Cooperation in Science and Technology (COST) project on electroporation-based technologies and treatments (9), in which electrical engineers, food scientists, and physicians have shared expertise and experience by interacting with each other in an open and respectful manner. As a result, pulsed electric field technologies and other high hydrostatic pressure technologies and equipment, developed by food scientists and engineers, are now being applied in medicine, cosmetics, pharmacy, and biotechnology (10, 11).

Interestingly, in 2014, Sharp pointed out that “Physicists gave engineers the electron and they created the IT revolution. Biologists gave engineers the gene and together they will create the future. Perhaps when the biologists give the genes to the food scientists and engineers together they can create sustainable foods and precision diets for individual longevity” (12).

There are many emerging research areas in which transdisciplinary research approaches may shift the existing paradigm. For example, there has been a resurgence of interest in the microbiome and health. Although food scientists have worked in this area for more than 100 years, as evidenced by the high number of related dairy products in the marketplace today, understanding of microbial–host interactions, modes of action, and mechanistic pathways in disease ecology and treatment can best be achieved through multidisciplinary approaches (13–18). Another example is the current need for new and additional sources of food raw materials, such as work on insects and insect proteins (19, 20) and exploration of marine chitin, the second most abundant organic compound on earth (21), for food, biologics, medicine, and pharmaceutical agents (22).

In addition, the development of new technologies, such as pulsed electric fields (23), will allow for the development of more effective permeable biological membranes and will enable researchers to take advantage of the vast amount of existing but mainly forgotten expertise and product developments in the fields of single-cell proteins (24) as well as plant protein concentrates (25–27).

What is evident is that with limitations in funding resources, it is important to work toward a future agenda in which transdisciplinary research becomes the norm and food science is better integrated in biomedical research. Food science research without borders is critically needed now!

## Disclaimer

The opinions expressed are those of the authors and may not reflect those of their affiliated organizations.

## Conflict of Interest Statement

The authors declare that the research was conducted in the absence of any commercial or financial relationships that could be construed as a potential conflict of interest.
